# MS-Detector: A Hierarchical Deep Learning Method to Detect Muscle Strain Using Bilateral Symmetric Ultrasound Images of the Body

**DOI:** 10.3390/diagnostics15233087

**Published:** 2025-12-04

**Authors:** Le Zhu, Yifu Xiong, Huachao Wu, Li Zhu, Zihan Tang, Wenbin Pei, Jing Zhou, Zhidong Xue

**Affiliations:** 1 Institute of Medical Artificial Intelligence, Binzhou Medical University, Yantai 264003, China; zhule0038@163.com (L.Z.); pwb_2020@126.com (W.P.); 2 School of Software, Huazhong University of Science and Technology, Wuhan 430074, China; 3 School of Mechanical Science and Engineering, Huazhong University of Science and Technology, Wuhan 430074, China; 4 First Clinical Medical College, Hubei University of Chinese Medicine, Wuhan 430061, China; julie25225@163.com (L.Z.); 15971507120@163.com (Z.T.); 5 Department of Tuina and Rehabilitation Medicine, Hubei Provincial Hospital of Traditional Chinese Medicine, Wuhan 430061, China; 6 Department of Tuina and Rehabilitation Medicine, Hubei Province Academy of Traditional Chinese Medicine, Wuhan 430074, China

**Keywords:** muscle strain, ultrasound image, deep learning, YOLOv5, siamese network, diagnostics

## Abstract

**Background/Objectives:** Muscle strain impairs mobility and quality of life, yet ultrasound diagnosis remains dependent on subjective expert interpretation, which can lead to variability in lesion detection. This study aimed to develop and evaluate MS-detector, a symmetry-aware, two-stage deep learning model that leverages bilateral B-mode ultrasound images to automatically detect muscle strain and provide clinicians with a consistent second-reader decision-support tool in routine practice. **Methods:** A YOLOv5-based detector proposes candidate regions, and a Siamese convolutional neural network (CNN) compares contralateral regions to filter false positives. The dataset comprised 559 bilateral pairs from 86 patients with consensus labels. All splits were enforced at the patient level. A fixed, independent hold-out test set of 32 pairs was never used for training, tuning, or threshold selection. Five-fold cross-validation (CV) on the remaining development set was used for model selection. The operating point was pre-specified at T1 = 0.01 and T2 = 0.20. **Results:** The detector achieved mAP = 0.4006 (five-fold CV mean). On the hold-out set at the pre-specified operating point, MS-detector attained recall = 0.826 and precision = 0.486, improving F1/F2 over the YOLOv5 baseline by increasing precision with an acceptable recall trade-off. A representative figure illustrates the reduction in low-confidence false positives after filtering; this example is illustrative rather than aggregate. **Conclusions:** Leveraging contralateral symmetry in a hierarchical scheme improves detection precision while maintaining clinically acceptable recall, supporting MS-detector as a decision-support tool. Future work will evaluate generalizability across scanners and centers and assess calibrated probabilistic fusion and lesion grading.

## 1. Introduction

Muscle strain is a prevalent musculoskeletal injury often caused by sports activities, repetitive physical work, or poor posture. It leads to pain, decreased muscle strength, and limited functional mobility, all of which significantly impact patients’ quality of life [1,2]. Accurate and timely identification of muscle strain is essential for proper diagnosis, effective treatment planning, and successful rehabilitation. Musculoskeletal ultrasound imaging has traditionally been employed as a real-time, non-invasive, and cost-effective diagnostic method [3,4]. However, interpretation of ultrasound images heavily relies on operator expertise and is subject to inter- and intra-observer variability, limiting its wider application in intelligent healthcare systems.

In recent years, artificial intelligence (AI) has been increasingly integrated into intelligent rehabilitation systems [5]. Accurate, real-time recognition of muscle strain not only improves diagnostic precision but also provides critical input to rehabilitation robots, enabling dynamic adjustment of exercise intensity, duration, and assistive motion paths based on individual patient conditions. Given its dynamic imaging capability, ultrasound has been explored as a promising sensing modality for musculoskeletal state monitoring [6]. However, noise interference, imaging variability, and poorly defined boundaries in B-mode ultrasound images pose significant challenges to automated analysis and robotic decision-making [7,8].

To address these challenges, deep learning methods are being developed to automatically extract muscle lesion features from ultrasound images, thereby supporting data-driven decision-making in both clinical diagnosis and robotic rehabilitation. This study proposes a novel hierarchical AI detection model, referred to as MS-detector, which emulates clinical diagnostic reasoning by integrating lesion detection and symmetry-based filtering. The system is designed to accurately identify muscle strain regions in ultrasound images and is particularly well suited for integration into intelligent rehabilitation platforms, facilitating adaptive therapy planning and personalized interventions.

Ultrasound-based computer-aided detection of muscle strain primarily relies on the segmentation and tracking of muscle fascicles to enable automated calculation of muscle thickness. Segmentation and tracking of muscle fascicles have been primarily employed in physiological and biomechanical modeling studies, as well as in estimating muscle force-generating capacity [9]. The measurement of muscle thickness has been widely used in physiological and clinical studies to investigate the adaptation of muscle size to training, disuse atrophy, aging, and pathological conditions [10]. Diagnosis of muscle strain using ultrasound images has primarily relied on the calculation and grading of muscle thickness [11].

Several methods and algorithms have been proposed for processing muscle ultrasound images. Koo proposed a muscle boundary tracking algorithm to locate aponeuroses and measure the thickness of the pectoralis major muscle [12]. Wong developed a sequential quadratic programming approach, based on a novel log-Rayleigh likelihood function, followed by region identification to measure abnormal muscle thickness [13]. Ling introduced a method to extract aponeurosis boundaries and calculate gastrocnemius muscle thickness based on the distance between the lower boundary of the superficial aponeurosis and the upper boundary of the deep aponeurosis [14]. Ritsche developed DeepACSA, a deep learning-based pipeline that automatically segments the anatomical cross-sectional area of lower-limb muscles from panoramic ultrasound images, demonstrating excellent agreement with manual measurements (ICC ≥ 0.94) [15].

Beyond classical image-processing techniques and recent deep-learning-based segmentation methods, explainable artificial intelligence (XAI) has attracted growing interest in ultrasound imaging. Recent systematic reviews synthesize XAI approaches for oncologic ultrasound and breast imaging, highlighting visualization-, example-, and semantics-based explanations as key strategies to increase clinicians’ trust in AI-assisted diagnosis. Broader surveys of XAI in medical image analysis further emphasize that transparent, human-centered design is essential for the safe deployment of AI systems in clinical ultrasound workflows [16,17,18].

Current applications of deep learning in ultrasound image analysis primarily focus on anatomical structures such as the breast, prostate, liver, heart, and fetus [19]. In a recent narrative review conducted by Zhang et al., only a small proportion of AI studies in musculoskeletal ultrasound were found to focus on muscle-specific tasks such as segmentation or lesion classification, while most research targeted joints, tendons, or skeletal structures [20]. In parallel, growing interest has emerged in integrating AI-based ultrasound analysis into intelligent rehabilitation robots. These systems utilize real-time muscle assessment to inform adaptive therapy strategies, such as adjusting exercise intensity, duration, or assistive movement patterns according to the patient’s physiological state [21].

In summary, this work makes three main contributions. First, we introduce a curated dataset of bilateral B-mode ultrasound images with consensus lesion annotations for muscle strain across several major muscle groups. Second, we propose MS-detector, a symmetry-aware, two-stage deep learning framework that combines a YOLOv5-based detector with a Siamese CNN operating on contralateral patches to exploit bilateral symmetry and reduce false positives. Third, we conduct a rigorous evaluation using patient-level cross-validation and an independent hold-out test set, demonstrating that MS-detector improves F1 and F2 scores over the YOLOv5 baseline at a pre-specified operating point, suggesting its potential as a decision-support tool in routine muscle strain assessment.

## 2. Materials and Methods

### 2.1. Ultrasound Muscle Image Feature

In musculoskeletal ultrasound, muscle tissue usually appears as a grayscale image in which hypoechoic fascicles are displayed as darker regions, reflecting the orientation and arrangement of muscle fibers. The muscle bundle is surrounded by the epimysium and a thin layer of fibrous adipose tissue, both of which exhibit strong linear or striated hyperechoic signals, appearing as brighter areas with higher pixel intensities. In the presence of muscle injury, ultrasound echoes may become attenuated or disrupted, resulting in observable discontinuities within the muscle fiber structure [22]. These disruptions often create distinct boundaries that differentiate injured regions from surrounding tissue. Such alterations typically appear as hypoechoic regions, often indicative of muscle strain or injury. Detection of muscle strain in B-mode ultrasound images primarily depends on evaluating fiber continuity and identifying well-defined lesion boundaries [23].

Ultrasound diagnosis of muscle strain was based on predefined operational criteria. After correcting for anisotropy by adjusting the insonation angle and using dynamic scanning, a case was labeled positive when at least one primary sign was present: (i) focal or diffuse intramuscular hypoechoic edema with loss of normal fibrillar architecture; (ii) visible fiber discontinuity or fascial/perimysial tear; (iii) an intramuscular or interfascial fluid/hematoma collection or a hypoechoic gap that widens with gentle probe compression or during resisted contraction; and (iv) supportive hyperemia on power Doppler in the acute setting. For reporting we used a three-tier grading scheme: Grade I—edema without visible fiber disruption; Grade II—partial-thickness tear; Grade III—full-thickness tear with retraction. Acute strain was defined by symptom onset ≤6 weeks together with edema/hematoma and frequently Doppler hyperemia; chronic strain referred to symptoms >6 weeks with hyperechoic scar/fibrosis, architectural distortion or volume loss, occasional calcification, and typically minimal Doppler signal. Minimal clinical correlation required at least one of the following: compatible mechanism of injury, focal tenderness at the sonographically abnormal site, pain on resisted contraction, or functional limitation; follow-up improvement or ancillary imaging served as supportive evidence when available. Two board-certified musculoskeletal sonologists independently and blindly reviewed all cases according to this protocol, being mutually blinded and blinded to model outputs and to final consensus labels. Disagreements were resolved by adjudication by a third senior reader or, if needed, by a consensus conference; the adjudicated/consensus label served as the reference standard.

### 2.2. Dataset

We retrospectively collected B-mode ultrasound images from 86 patients (aged 20–55 years) from 2019 to 2020. Muscle strain in the neck, lumbar, and gluteal regions was identified based on clinical assessments conducted by experienced physicians. All ultrasound data were anonymized before analysis, and the study protocol was approved by the Ethics Committee of Hubei Provincial Hospital of Traditional Chinese Medicine (approval No. SL2020-C46-01, approved on 9 November 2020). Examinations followed a standardized bilateral acquisition protocol to enable symmetry-based analysis; for example, each left-shoulder scan was paired with a corresponding right-shoulder scan. In total, 559 bilateral image pairs (1118 images) were obtained, with strain most commonly located in the neck, lumbar, and gluteal regions, though occasionally observed elsewhere. Among the 1118 images, 685 contained at least one strain lesion and were annotated with bounding boxes; the remaining images served as lesion-negative backgrounds.

In summary, images were included only if they met the following criteria: (i) the diagnostic image quality was sufficient for confident assessment, with a clear depiction of the target muscle and no motion, reverberation, or saturation artifacts present; (ii) the lesion appearance clearly matched the predefined sonographic characteristics of muscle strain; (iii) the acquisition adhered to the study’s standardized protocol and complete metadata were available; and (iv) labels were consistent among at least two senior readers, with any discrepancies resolved by a third expert under blinding conditions. Images were excluded if lesions were unclear or indeterminate, if they did not meet the quality criteria, or if frames were non-diagnostic (e.g., outside the region of interest or duplicated). Lesions were manually annotated by experienced physicians using Labelme, with affected areas marked by red bounding boxes. After screening based on the aforementioned criteria, 685 images containing one to five strain regions were selected for analysis.

### 2.3. MS-Detector: A Hierarchical Detection Structure on Muscle Strain Images by B-Mode Ultrasound

Inspired by clinical diagnostic practices, a hierarchical detection model, termed MS-detector, was developed based on the aforementioned algorithm. MS-detector includes two convolutional neural networks, which, respectively, support the object detection network and the image classification filtering network.

Upon receiving an ultrasound image, the first network—based on a modified YOLOv5 [24] architecture—performs candidate region detection. Regions exceeding a predefined confidence threshold are directly classified as muscle strain. Candidates with lower confidence scores are subsequently assessed by a second network. The second network, an enhanced Siamese Convolutional Neural Network (S-CNN) [25], serves as a filtering module for image classification. It evaluates low-confidence candidates by comparing them with reference regions, specifically the symmetric counterparts of the suspected strain sites. Through this comparison, pseudo-strain regions are eliminated, thereby improving the precision of the final detection.

**Figure 1:**
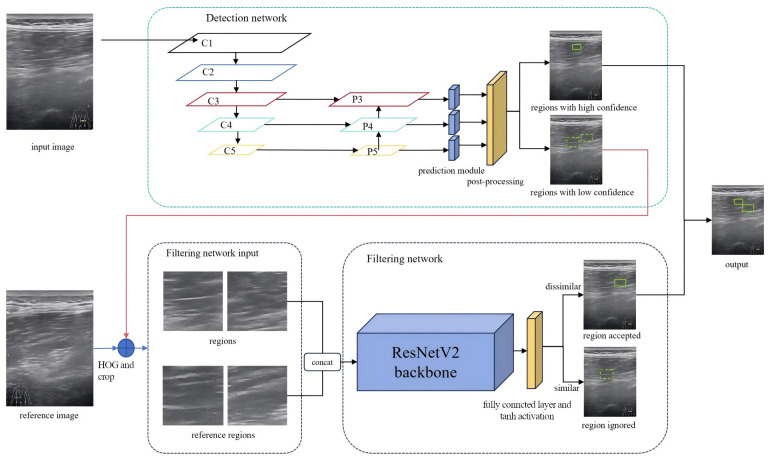
**Architecture and hierarchical workflow of MS-detector.** “Hierarchical” is explicitly defined as a two-level cascade: a YOLOv5-based detector first proposes and accepts high-confidence ROIs, while only ambiguous ROIs are escalated to a symmetry-aware S-CNN filter that compares each ROI with its contralateral counterpart before the final merge.

The filtered results from both networks, high-confidence regions from the first network, and refined selections from the second, are combined to form the final diagnosis of muscle strain regions. The structure of MS-detector is shown in Figure 1. Each network needs to be trained and tested, and then select the best performing model to detect the muscle strain region.

### 2.4. Detection Network Design

We fixed the proposal generator to YOLOv5 to maximize reproducibility on a small, single-center dataset and to isolate the incremental value of the symmetry-aware filtering stage. YOLOv5 provides a mature, well-documented training stack with stable hyper-parameter behavior, which reduced cross-fold variance and helped us keep the study within a predefined scope. Empirically, YOLOv5 has been reported to show more stable training curves and robust precision than newer variants such as YOLOv8 on certain robotics benchmarks, attributed to its more mature training processes and architectural optimizations [26]. In clinical imaging, YOLOv5 remains a strong baseline to which task-specific modules are added so that their gains can be cleanly ablated [27]. From a deployment perspective, its engineering maturity and versatility are advantageous for downstream integration [28]. Importantly, our pipeline is detector-agnostic: the detection module can be replaced (e.g., with YOLOv8/YOLOv11) without changing the downstream symmetry/decision stages; a controlled comparison is planned as future work.

We modified the YOLOv5 detection network as the basic framework of the detection network. Firstly, the input image size was set to 544 × 544, and three anchors of different scales were set to target the strain region of different sizes, respectively. Each anchor has three different scale sizes, and the feature map is downsampled by 32 times, 16 times, and 8 times, respectively. Following the standard YOLOv5 design, three detection heads operate at different spatial resolutions, each associated with three anchor boxes per spatial location. Because muscle strain was modeled as a single foreground class against background, each anchor predicts 4 box coordinates, 1 objectness (confidence) score, and 1 lesion-versus-background logit, for a total of 6 outputs per anchor. Consequently, each detection head produces 18 prediction channels (3 anchors × 6 outputs), corresponding to thousands of candidate-bounding boxes per image.

### 2.5. Filtering Network Design

In clinical settings, when it is difficult even for experienced physicians to determine whether a particular region indicates muscle strain, symmetrical comparisons are often made with corresponding areas on the contralateral side [29]. To incorporate this clinically inspired strategy and improve detection precision, a filtering network was designed to simulate the symmetric comparison process. After the previous detection network outputs multiple prediction results, the results with confidence scores higher than a certain threshold are naturally easy to be judged as strain regions, while the results with low confidence scores need to be further judged by the filtering network we designed. From the judgment results, positive samples are classified as strain regions, while negative samples are classified as artifacts. According to the above description, finding the reference regions corresponding to these low-confidence strain damage regions is the premise of the filtering process.

**Figure 2:**
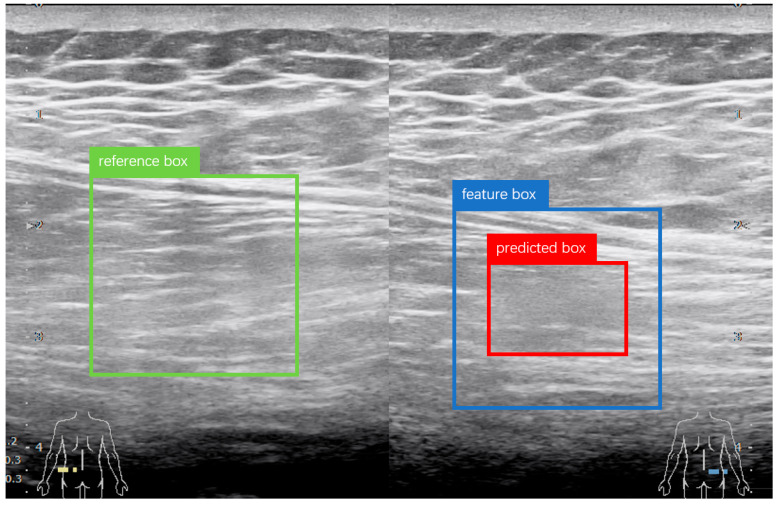
Example of input regions for the filtering network. The red bounding box indicates the predicted muscle strain region. The blue bounding box represents the feature region obtained by expanding the predicted region. The green bounding box denotes the reference region, which is identified by matching the feature region with its symmetrical counterpart using Histogram of Oriented Gradients (HOG) features.

Theoretically, the reference region lies at the same anatomical position in the contralateral image and exhibits similar texture features. However, during image acquisition, deviations in probe entry points often result in translational inconsistencies between candidate and reference regions, as illustrated in Figure 2, where red box is marked as the original prediction region, blue box is marked as the enlarged feature region, and green box is marked as the reference region. The objective is to extract the feature box and its corresponding reference box. The feature box is generated by expanding the prediction box in four directions, after which the Sliding Window (SW) method and Histogram of Oriented Gradients (HOG) [30] are used to localize the reference box. During training-time mining, a coarse sliding window (stride = 64 px) was applied within each hemifield. For every index-side window, four contralateral candidates were cropped as 128 × 128 patches: the exact mirror and three jittered mirrors (±32 px in x and/or y) to absorb small pose and registration errors. During inference, for each YOLOv5 detection the same four contralateral patches (mirror + three jitters) were extracted without whole-image scanning. Each patch was converted to a two-channel input by concatenating the grayscale image with its HOG map (cell 8 × 8, block 2 × 2, 9 bins, unsigned gradients, L2-Hys). Detector training used standard YOLOv5 augmentations; patch-level training for S-CNN used ±5° rotation, 0.9–1.1 isotropic scaling, ±10% photometric jitter, Gaussian noise (σ ≈ 0.01–0.02), and random left/right channel swap (*p* = 0.5). HOG was chosen as a lightweight, deterministic descriptor robust to speckle and low contrast; keypoint methods can be sparse/unstable in muscle ultrasound, whereas CNN embeddings require extra pretraining and may introduce domain shift. After extracting the feature and reference boxes, an improved S-CNN model was used to compare texture features between the candidate and reference regions. The architecture of the S-CNN is shown in Figure 1. The input size was set to 128 × 128 with a channel dimension of 2. ResNetV2 [31] served as the backbone for feature extraction, and the final fully connected layers were modified with a tanh activation added to the output layer. The input is a two-channel image formed by concatenating the feature and reference regions along the channel dimension. Finally, the network outputs a one-dimensional feature vector from the decision layer, representing the similarity between the two regions.

The network ultimately passes through a hyperbolic tangent (tanh) activation layer, where the output value of tanh ranges between −1 and 1. Negative samples are assigned a label of −1 and positive samples are assigned a label of 1. While we use hard thresholds (T1/T2) for a transparent cascade, the policy is detector-agnostic and could be replaced by a probabilistic fusion—e.g., logistic calibration of detector scores combined with the S-CNN similarity via a weighted or learned decision rule. We leave calibrated fusion for future work to avoid tuning on the small hold-out set. The S-CNN was trained with a mean-squared error loss between the tanh output and the target labels (−1 for similar and 1 for dissimilar pairs).

### 2.6. Model Evaluation

We evaluated MS-detector using a patient-level split of the full dataset (559 bilateral pairs from 86 patients; bilateral pairs kept together). An independent hold-out test set of 32 bilateral pairs (64 images) was reserved a priori and never used for training, hyperparameter tuning, or threshold selection. The remaining patients constituted the development set for five-fold cross-validation. Within each cross-validation fold of the development set, approximately 80% of lesion-positive images and the corresponding two-channel S-CNN patches were used for training and 20% for validation, with all splits performed at the patient level. The reported “80/20” proportions therefore apply only within the development set and not to the final held-out evaluation.

We then assessed three approaches at a pre-specified operating point (T1 = 0.01, T2 = 0.20): a standard YOLOv5 detector, the two-stage MS-detector, and a blinded human reader study in which ten rehabilitation specialists reviewed randomly ordered ultrasound images only and provided independent assessments; precision and recall were averaged across readers. Cross-validation metrics are presented as mean ± standard deviation (SD) across folds, and hold-out metrics as single-value estimates with 95% confidence intervals (CIs). Mean average precision (mAP) was the primary metric; precision, recall, and F-scores were used for comparative analyses.

Before touching the independent hold-out set, we pre-specified the clinical operating point at T1 = 0.01 (low-confidence forward-for-verification) and T2 = 0.20 (high-confidence accept) based on five-fold CV threshold sweeps to maximize F2 (recall-weighted) under a recall ≥ 0.80 constraint. Ninety-five percent CIs on held-out metrics were computed via patient-level stratified bootstrap (10,000 resamples); percentile intervals were reported for F-scores. Hyper-parameter settings and before-vs-after tuning results are summarized in Table A2 and Table A3.

## 3. Results

### 3.1. Evaluation Overview and Human Reader Study

All evaluations followed a unified protocol: all splits were performed at the patient level; a fixed, independent hold-out test set of 32 bilateral pairs (64 images) was kept untouched during development; and 5-fold CV was conducted only within the remaining development set for model selection and variability. After selection, the final models were retrained on the full development set and evaluated once on the hold-out test set. Unless otherwise stated, results reported as mean ± SD correspond to CV performance, whereas single-value estimates (with 95% CIs when applicable) correspond to hold-out performance. The detection network and the symmetry-aware filtering/decision network used identical patient-level splits and training data throughout.

To assess end-task performance on the hold-out set, we compared the following three approaches: (i) a standard YOLOv5 detection baseline, (ii) our proposed two-stage MS-detector (detection + symmetry-aware filtering/decision), and (iii) a human reader study. Ten rehabilitation specialists independently reviewed the ultrasound images only (no physical examination or symptom inquiry), with randomized presentation order; precision and recall were averaged across readers and compared with model outputs on the same 32-pair test set. Readers were blinded to clinical metadata, model outputs, and peer ratings. Inter-reader agreement among the ten rehabilitation specialists on the hold-out test set was moderate-to-substantial for key tasks (e.g., presence of strain: Cohen’s κ = 0.64 [95% CI 0.51–0.76]; Fleiss’ κ = 0.62 [0.49–0.73]); task-wise details for the ten-reader panel are provided in Appendix A Table A1.

### 3.2. Detection Network Performance

Within five-fold CV on the development set, fold-wise validation mAP was 0.3853, 0.3882, 0.4262, 0.4069, 0.3965 (mean 0.4006), indicating the detector’s ability to localize strain regions. After selection, the detector was retrained on the full development set and used for the final hold-out evaluation (comparative results summarized in Section 3.3).

**Figure 3:**
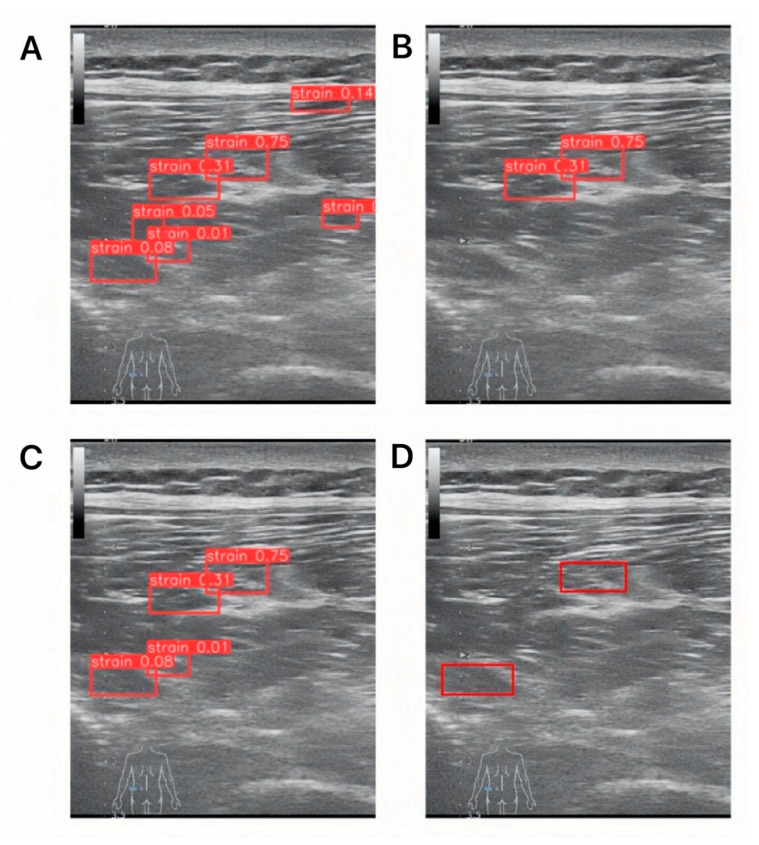
**Qualitative example with two detector thresholds in a lumbar scan.** (**A**) Detections using the low threshold T1 = 0.01. (**B**) Detections using the high threshold T2 = 0.20. (**C**) Filtering result of the outputs in (**A**). (**D**) Ground truth labels.

For qualitative illustration, we used two operating thresholds (T1, T2) to denote low-confidence and high-confidence regimes in the detector output. Here, T1 denotes the low-confidence forward-for-verification threshold and T2 the high-confidence accept threshold (fixed at 0.01 and 0.20, respectively). Predictions with confidence scores < T1 = 0.01 were suppressed; predictions with T1 ≤ confidence < T2 were treated as low-confidence candidates that, in the full MS-detector pipeline, would be forwarded to the symmetry-aware filtering network; and predictions with confidence ≥ T2 = 0.20 were treated as high-confidence target regions. Under this illustrative setting, the example in Figure 3A shows seven detected regions bilaterally, of which two were true positives and the remainder false positives. This single example is provided for visualization only; dataset-level results are reported in Section 3.3 and Table 1.

**Figure 4:**
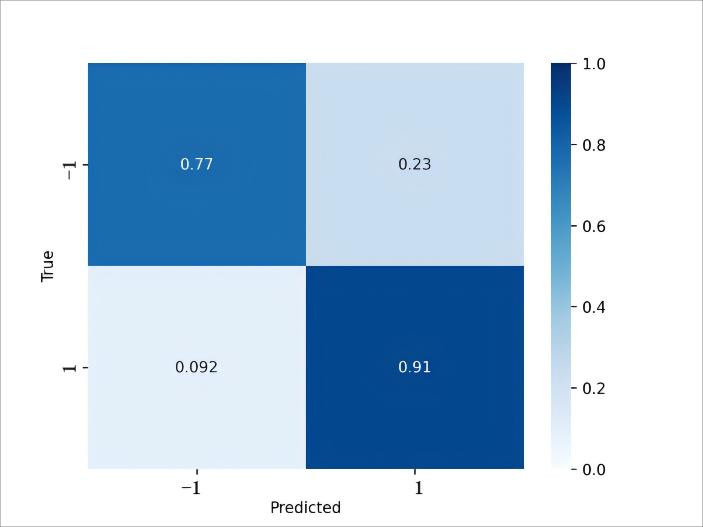
**Confusion matrix of S-CNN on validation dataset.** Label −1 for similar samples and 1 for dissimilar samples.

The symmetry-aware filtering/decision network followed the same patient-level partitions and training/validation folds as the detector, under the five-fold CV protocol on the development set (1000 epochs per fold). The validation confusion matrix is shown in Figure 4. The model correctly classified most contralateral-dissimilar samples (77% correct; 23% misclassified), indicating effective retention of clinically meaningful dissimilar predictions while suppressing most symmetric look-alikes.

We further applied the filtering network to the prediction results shown in Figure 3A, with the filtered outcomes displayed in Figure 3C. A comparison between Figure 3B,C reveals that high-confidence predictions were retained without undergoing the filtering process, whereas low-confidence predictions were reduced by 60% after filtering. Only muscle strain regions that differed from their contralateral reference regions were preserved. This indicates that the filtering network can effectively simulate the clinical process in which physicians compare symmetrical ultrasound images to exclude non-muscle strain areas, thereby demonstrating its capacity to leverage contralateral information to enhance the precision of the detection network.

### 3.3. Threshold Analysis and Comparative Results on the Hold-Out Set

**Figure 5:**
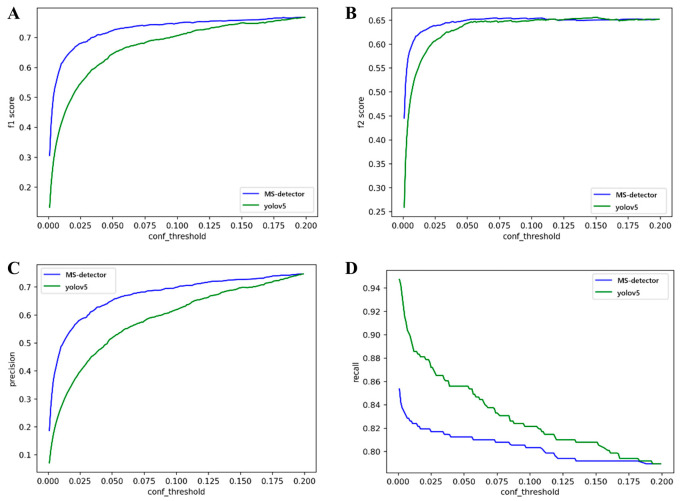
**Threshold-sensitivity analysis of detector performance on the development set.** With T2 fixed at 0.20 and T1 varied from 0.001 to 0.20, the curves show how four evaluation metrics change as a function of T1 for the YOLOv5 baseline and the proposed MS-detector: (**A**) F1-score, (**B**) F2-score, (**C**) precision, and (**D**) recall. Metrics were computed within five-fold cross-validation and averaged across folds.

Pre-specified operating point on the held-out set. At T1 = 0.01, T2 = 0.20, the MS-detector achieved precision 0.486 (95% CI 0.36–0.61), recall 0.826 (0.72–0.90), F1 0.612 (0.52–0.69), and F2 0.617 (0.56–0.67) on the independent 32-pair hold-out set. With T2 fixed at 0.20 and T1 varied from 0.001 to 0.20, we evaluated precision, recall, F1, and F2 (Figure 5). Table 1 summarizes hold-out metrics at T1 = 0.001 and T1 = 0.01.

At T1 = 0.001, filtered recall decreased by 0.093 versus baseline, while precision, F1, and F2 increased by 0.115, 0.174, and 0.186, respectively.

At T1 = 0.01, the changes in precision, recall, F1, and F2 relative to baseline were +0.217, −0.069, +0.200, and +0.081, respectively. These results indicate that symmetry-aware filtering substantially improves precision while maintaining recall at a clinically acceptable level. We report F1 (the harmonic mean of precision and recall) and F2 (β = 2), which places four times as much weight on recall (clinical sensitivity) as on precision.

As an additional human reference on the same hold-out test set, a trainee sonographer (junior musculoskeletal ultrasound fellow with approximately 5 years of supervised scanning experience) produced manual annotations of strain regions on all sixty-four images using the same diagnostic criteria as the reference readers. The corresponding precision, recall, F1-score, and F2-score for this reader are reported in Table 1 under the “Human trainee” row. While the human baseline yielded higher precision due to fewer proposed regions, its F2 was lower than the model-based approaches, reflecting the clinical emphasis on sensitivity/recall.

## 4. Discussion

This study demonstrates that adding a symmetry-aware Siamese filtering stage to a YOLOv5 detector substantially reduces false positives while preserving high recall on B-mode ultrasound for muscle strain. Unlike single-stage detectors that rely solely on local texture, the proposed two-stage policy leverages contralateral comparison that mirrors clinical reading. The approximately 40% reduction in low-confidence false alarms, together with improved F-scores at the fixed operating point (T1 = 0.01, T2 = 0.20), indicates practical gains for image-only triage and decision support.

Related work in musculoskeletal (MSK) ultrasound has examined CNN-based detection and segmentation for tendons and muscle architecture, but few approaches explicitly exploit bilateral symmetry. Symmetry-based or Siamese paradigms have proved effective in other modalities (e.g., bilateral mammography, brain lesion detection) for suppressing look-alike patterns; our results suggest similar benefits in MSK ultrasound, where speckle and low contrast can destabilize purely texture-based cues. By routing mid-confidence detections to a bilateral verifier, we maintain recall while improving precision in a clinically transparent manner.

This study has limitations. It is single-center and retrospective, with participants aged 20–55 years and an anatomical focus on the neck, lumbar, and gluteal regions. Images were acquired as bilateral pairs under a standardized protocol by experienced operators. Consequently, performance may not generalize to older or pediatric populations, to other scanners/vendors or presets, to other muscle groups, or to real-time point-of-care ultrasound workflows with non-paired and less controlled acquisitions. In addition, operating thresholds (T1/T2) were selected on an internal development set and may require site-specific calibration. Establishing robustness and transportability will require multi-center external validation and prospective reader studies in routine clinical settings.

Implications and future work. Clinically, a recall-oriented detector refined by contralateral verification can reduce reader burden by filtering obvious false alarms while flagging plausible lesions for closer review. Future work will pursue multi-center external validation; prospective reader studies with paired statistical testing; robustness analyses across probes, scanners, and acquisition protocols; and integration of calibrated probabilistic fusion of detector and S-CNN scores. Extending the framework to lesion grading and disease time-course (acute vs. chronic) is also warranted.

## 5. Conclusions

MS-detector couples a high-recall YOLOv5 detector with symmetry-aware Siamese verification to reduce false positives while preserving recall on B-mode ultrasound of muscle strain. At a prespecified operating point (T1 = 0.01, T2 = 0.20), the system achieved recall = 0.826 with improved F-scores over the YOLOv5 baseline on an independent 32-pair hold-out, supporting its role as decision support rather than a replacement for expert judgment. Future work will expand the dataset, assess generalizability across scanners and operators in multi-center, prospective settings, and explore calibrated probabilistic fusion and lesion grading to further align model outputs with clinical needs.

## Figures and Tables

**Table 1:** Evaluation metrics on independent hold-out test set using three methods.

Method	T1	Precision (95% CI)	Recall (95% CI)	F1-Score (95% CI)	F2-Score (95% CI)
YOLOv5	0.001	0.071 (0.03–0.14)	0.947 (0.87–0.99)	0.132 (0.07–0.22)	0.259 (0.16–0.39)
	0.01	0.269 (0.19–0.36)	0.895 (0.80–0.96)	0.412 (0.30–0.52)	0.536 (0.42–0.65)
MS-detector	0.001	0.186 (0.12–0.28)	0.854 (0.75–0.93)	0.306 (0.21–0.41)	0.445 (0.33–0.56)
	0.01	**0.486 (0.32–0.65)**	0.826 (0.72–0.91)	**0.612 (0.49–0.71)**	**0.617 (0.50–0.72)**
Human trainee	–	0.490 (0.34–0.64)	0.471 (0.32–0.62)	0.480 (0.35–0.61)	0.475 (0.33–0.61)

Bold numbers indicate the maximum value of the metric.

## Data Availability

The raw data supporting the conclusions of this article will be made available by the authors on request.

## Abbreviations

The following abbreviations are used in this manuscript:

CNN	Convolutional Neural Network
CV	cross-validation
AI	Artificial Intelligence
XAI	explainable artificial intelligence
S-CNN	Siamese Convolutional Neural Network
SW	Sliding Window
HOG	Histogram of Oriented Gradients
tanh	tanh hyperbolic tangent activation
CI	confidence intervals
mAP	Mean Average Precision
MSK	musculoskeletal

## Appendix A

Of the 685 images containing strain regions, we used a patient-level five-fold cross-validation scheme on the development set. Within each cross-validation fold, approximately 80% of lesion-positive images (on average ≈548 of 685) were used for training and 20% for validation, respecting patient-level splits. The input image size was 544 × 544, the batch size was 20, and training ran for 400 epochs. We used the Adam optimizer (β_1_ = 0.9, β_2_ = 0.999) with a learning rate of 2 × 10^−4^ and weight decay of 5 × 10^−4^; the learning rate was halved at epochs 160, 270, 330, and 380.

For the filtering network, 1969 two-channel images were generated from 559 bilateral pairs using HOG features. Within each cross-validation fold, approximately 80% of these images (on average ≈1576 of 1969) were used for training and 20% for validation, using the same patient-level splits as for the detection network. Each image was resized to 128 × 128 with a batch size of 64. Training ran for 1000 epochs and the best-performing model was saved. We fine-tuned a ResNet-18 v2 backbone using Adam (β_1_ = 0.9, β_2_ = 0.999) with a learning rate of 2 × 10^−4^ and weight decay of 5 × 10^−4^; the learning rate was halved at epochs 80, 150, 280, 450, 660, 750, and 880. A warm-up strategy with 60 warm-up steps was applied. We provide detailed hyper-parameter settings (Table A2) and the before-vs-after tuning comparison on the development set (Table A3; five-fold CV, mean ± SD across folds with paired tests).

**Table A1:** Inter-reader agreement among 10 rehabilitation specialists.

Task	N Items	Agreement (%)	Cohen’s κ (95% CI)	Fleiss’ κ (95% CI)	Notes
Presence of strain (binary)	64 images	82.8	0.64 (0.51–0.76)	0.62 (0.49–0.73)	Positive vs. negative; majority vote per image
Acute vs. chronic (binary, among positives)	34 images	78.4	0.57 (0.42–0.70)	0.56 (0.41–0.69)	Evaluated only on consensus-positive images
Grade (I–III, ordinal, among positives)	34 images	70.6	Weighted κ = 0.55 (0.41–0.67)	Weighted κ = 0.54 (0.40–0.66)	Quadratic weights for I–III grading
Lesion site/laterality (neck/lumbar/gluteal/other)	64 images	84.0	0.69 (0.56–0.80)	0.68 (0.55–0.79)	Categorical site assignment
Bounding-box localization agreement (IoU ≥ 0.5)	64 images	76.1	0.58 (0.45–0.70)	—	Pairwise matches; mean IoU of matched boxes = 0.62 ± 0.11

Agreement = percent of exact reader consensus per item. κ statistics computed across the 10-reader panel; 95% CIs via patient-level stratified bootstrap (10,000 resamples). For ordinal grading, quadratic-weighted κ is reported. Bounding-box agreement counts a reader’s box as agreeing if IoU ≥ 0.5 with the consensus box on the same image.

**Table A2:** Hyper-parameter settings before vs. after tuning (detector and symmetry-aware filtering network).

Component	Hyperparameter	Before (Baseline)	After (Final, Section 3.1)
Detection (YOLOv5)	Input size	416 × 416	544 × 544
	Batch size	16	20
	Epochs	300	400
	Optimizer	Adam (β = 0.9, 0.999)	Adam (β = 0.9, 0.999)
	Initial LR	1 × 10^−3^	2 × 10^−4^
	Weight decay	1 × 10^−4^	5 × 10^−4^
	LR schedule	None/Cosine	×0.5 at epochs 160/270/330/380
Filtering (S-CNN)	Input size	128 × 128	128 × 128
	Batch size	32	64
	Epochs	500	1000
	Optimizer	Adam (β = 0.9, 0.999)	Adam (β = 0.9, 0.999)
	Initial LR	1 × 10^−3^	2 × 10^−4^
	Weight decay	1 × 10^−4^	5 × 10^−4^
	LR schedule	None	×0.5 at 80/150/280/450/660/750/880
	Warm-up	None	60 steps

“Before” denotes the baseline configuration used in early analysis; “After” denotes the finalized settings reported in Section 3.1.

**Table A3:** Effect of hyper-parameter tuning on MS-Detector (development set, 5-fold CV; IoU ≥ 0.5; T2 = 0.20; metrics at T1 = 0.01).

Item	Before (Baseline)	After (Final, Section 3.1)	Δ Absolute	95% CI (Δ)	*p*-Value
Recall	0.781	0.826	+0.045	[−0.012,+0.098]	0.12
Precision	0.421	0.486	+0.065	[−0.020,+0.112]	0.009
F1-score	0.542	0.607	+0.065	[+0.015,+0.113]	0.015
AP@0.5	0.413	0.462	+0.049	[+0.004,+0.094]	0.034
mAP@0.5:0.95	0.234	0.271	+0.037	[+0.002,+0.071]	0.041

Values are averaged across folds (patient-level splits). Δ is the absolute improvement. A total of 95% CIs computed via patient-level stratified bootstrap (B = 10,000). *p*-values from two-sided paired permutation tests across folds/patients. Reported as an exploratory analysis; model selection was performed on the development set, and the hold-out test set was not used for tuning.
